# Resting Heart Rate and the Risk of Microvascular Complications in Patients With Type 2 Diabetes Mellitus

**DOI:** 10.1161/JAHA.112.002832

**Published:** 2012-10-25

**Authors:** Graham S. Hillis, Jun Hata, Mark Woodward, Vlado Perkovic, Hisatomi Arima, Clara K. Chow, Sophia Zoungas, Anushka Patel, Neil R. Poulter, Giuseppe Mancia, Bryan Williams, John Chalmers

**Affiliations:** The George Institute for Global Health, University of Sydney, Australia (G.S.H., J.H., M.W., V.P., H.A., C.K.C., S.Z., A.P., J.C.); Imperial College, London, United Kingdom (N.R.P.); University College London, London, United Kingdom (B.W.); University of Milano-Bicocca, Milano, Italy (G.M.)

**Keywords:** diabetes mellitus, type 2, heart rate, microcirculation

## Abstract

**Background:**

A higher resting heart rate is associated with an increased probability of cardiovascular complications and premature death in patients with type 2 diabetes mellitus. The impact of heart rate on the risk of developing microvascular complications, such as diabetic retinopathy and nephropathy, is, however, unknown. The present study tests the hypothesis that a higher resting heart rate is associated with an increased incidence and a greater progression of microvascular complications in patients with type 2 diabetes mellitus.

**Methods and Results:**

The relation between baseline resting heart rate and the development of a major microvascular event was examined in 11 140 patients who participated in the Action in Diabetes and Vascular Disease: Preterax and Diamicron Modified Release Controlled Evaluation (ADVANCE) study. Major microvascular events were defined as a composite of new or worsening nephropathy or new or worsening retinopathy. Patients with a higher baseline heart rate were at increased risk of a new major microvascular complication during follow-up (adjusted hazard ratio: 1.13 per 10 beats per minute; 95% confidence interval: 1.07–1.20; *P*<0.001). The excess hazard was evident for both nephropathy (adjusted hazard ratio: 1.16 per 10 beats per minute; 95% confidence interval: 1.08–1.25) and retinopathy (adjusted hazard ratio: 1.11 per 10 beats per minute; 95% confidence interval: 1.02–1.21).

**Conclusion:**

Patients with type 2 diabetes mellitus who have a higher resting heart rate experience a greater incidence of new-onset or progressive nephropathy and retinopathy.

**Clinical Trial Registration:**

URL: http://www.clinicaltrials.gov. Unique identifier: NCT00145925. http://www.advance-trial.com/static/html/prehome/prehome.asp

## Introduction

Microalbuminuria and retinopathy are indicators of microvascular dysfunction, and both predict a poorer outcome in patients with diabetes^1–3^ and in individuals with normal glycemic control.^4,5^ In particular, even mild degrees of microalbuminuria are a strong and independent predictor of major cardiac events in the general population^6,7^ and among patients with diabetes^4^ and hypertension.^8^ Likewise, several studies have reported an association between a higher resting heart rate and an increased risk of cardiovascular complications in unselected populations and in patients with cardiac disease.^9,10^ Recently, we reported a similar relation between resting heart rate and all-cause death, cardiovascular death, and major cardiovascular events in patients with type 2 diabetes mellitus.^11^ This was not attenuated after adjustment for other factors that influence heart rate and outcome.^11^

Although a growing volume of data supports an association between higher resting heart rate and a greater risk of macrovascular events in patients with type 2 diabetes, the impact of heart rate on the risk of developing microvascular complications, such as diabetic retinopathy and nephropathy, is unknown. There are, however, reasons to suspect that an elevated resting heart rate might predict microvascular outcomes in this setting. In patients with hypertension, a higher resting heart rate has been associated with a greater prevalence of microalbuminuria,^12,13^ a relation that was maintained after correction for a variety of potential confounding factors. Similarly, a weak, but significant and independent, association between baseline resting heart rate and urinary albumin:creatinine ratio (ACR) was recently reported in the Randomised Olmesartan and Diabetes Microalbuminuria Prevention study.^14^ However, not all existing data support an association between prevalent heart rate and albuminuria,^15^ and the cross-sectional results that are currently available cannot address the relation between heart rate and incident or progressive microalbuminuria.

A higher resting heart rate has been reported in patients with diabetic retinopathy^16^ and could be due to the relation between retinopathy and autonomic dysfunction. Once again, however, the importance of heart rate in the development or progression of diabetic eye disease remains unclear. Using data from the Action in Diabetes and Vascular Disease: Preterax and Diamicron Modified Release Controlled Evaluation (ADVANCE) study (http://ClinicalTrials.gov No. NCT00145925), we tested the hypothesis that a higher resting heart rate would be associated with both an increased incidence and a greater progression of microvascular complications in patients with type 2 diabetes mellitus.

## Methods

The ADVANCE study recruited 11 140 patients with type 2 diabetes mellitus from 215 centers in 20 countries between June 2001 and March 2003.^17,18^ The study made 2 randomized comparisons: (1) a double-blind assessment of the efficacy of a fixed-combination perindopril–indapamide (2 mg/0.625 mg for 3 months, increasing, if tolerated, to 4 mg/1.25 mg) versus placebo and (2) an open-label evaluation of an intensive glucose-lowering regimen with modified-release gliclazide, with a target hemoglobin A1c of ≤6.5%, versus standard guideline-based glycemic control. The study was approved by the local ethics committee for each participating center, and all participants provided written informed consent. Participants were ≥55 years of age and had been diagnosed with type 2 diabetes mellitus after the age of 30 years. In addition, they were required to have a history of cardiovascular disease or ≥1 additional cardiovascular risk factor.^19^ Detailed study methods^19^ and the main results^17,18^ of the ADVANCE study have been reported previously.

Heart rate was measured at the same time as blood pressure with a digital monitor (Omron HEM-705CP, Omron Healthcare Inc, Lake Forest, IL). Recordings were made after the patient had been resting in a seated position for a minimum of 5 minutes. Three readings were taken, and the mean of the latter 2 was used. Weight, height, urinary ACR, serum creatinine, fasting lipid levels, and glycosylated hemoglobin also were measured at baseline.

The present study assesses the relation between resting heart rate and the development of a major microvascular event (a coprimary endpoint of the ADVANCE study) over the length of the trial (median follow-up of 4.4 years). Major microvascular events were defined, a priori, as a composite of new or worsening nephropathy or retinopathy. The former was defined as ≥1 of the following: the development of macroalbuminuria, defined as a urinary ACR of >300 μg albumin per milligram of creatinine (confirmed by 2 positive results); doubling of the serum creatinine level to ≥200 μmol/L (unless in the context of acute illness, with subsequent recovery of renal function, or in the context of the terminal phase of illness); the need for renal replacement therapy due to diabetic kidney disease, in the absence of another acute medical cause that could require transient dialysis; or death due to renal disease. Participants had their creatinine levels measured as part of the study protocol at baseline, 4 months, and 1 year and annually thereafter until completion of the study. Renal function was checked out with these time points at the discretion of their treating clinicians. Urinary ACR was measured locally as part of the study protocol at baseline, 2 years, 4 years, and completion of the study. New or worsening retinopathy was defined as ≥1 of the following: the development of proliferative retinopathy (identified by the incidence of new blood vessels on the disc or elsewhere, vitreous hemorrhage, preretinal hemorrhage, fibrous proliferations on the disc or elsewhere in a participant known not to have this condition at entry); macular edema (characterized by a retinal thickening within 1 disc diameter of the macular center, in a participant known not to have this condition at entry); diabetes-related blindness (corrected visual acuity 3/60 or worse, persisting for ≥3 months and known to not be due to nondiabetic causes, in a participant known not to have this condition at entry); or the use of retinal photocoagulation therapy. Participants underwent formal eye examination and visual acuity testing at baseline, 2 years, 4 years, and completion of the study.

Secondary endpoints were the individual components of the primary outcome (new or worsening nephropathy and new or worsening retinopathy). An Endpoint Adjudication Committee, unaware of treatment allocation, reviewed source documentation for all individuals who had a suspected primary endpoint of the ADVANCE study. This included all the microvascular endpoints considered in the present analyses.

### Statistical Analyses

Baseline resting heart rate was considered both as a continuous variable and after grouping into approximately equal fifths according to its quintiles. Trends in baseline characteristics across the fifths of heart rate were compared with linear and logistic regression. Cox regression analyses were used to calculate hazard ratios with 95% confidence intervals. These were performed in models adjusted for age, sex, ADVANCE study blood pressure treatment arm, and ADVANCE study glycemic control arm and in models adjusted for multiple baseline covariates (age, sex, ADVANCE study randomized treatment, body mass index, duration of diabetes, glycosylated hemoglobin, estimated glomerular filtration rate, urinary ACR, systolic blood pressure, diastolic blood pressure, history of a prior macrovascular complication of diabetes [myocardial infarction, stroke, hospital admission for a transient ischemic attack, hospital admission for unstable angina, coronary revascularization, peripheral revascularization or amputation secondary to peripheral vascular disease], history of hospitalization for heart failure, participation in moderate or vigorous exercise for >15 minutes at least once weekly, current cigarette smoking, total cholesterol, triglycerides, atrial fibrillation, treatment with calcium channel blockers, and treatment with β-blockers). Interactions were tested by adding appropriate terms to Cox models. Linearity was assessed by comparing the categorical model with the linear trend model.^20^ It is possible that death during follow-up might affect the association between heart rate and the outcomes of interest. Therefore, we estimated subdistribution hazard ratios after correcting for the competing risk of death according to the methods of Fine and Gray.^21^ Odds ratios and 95% confidence intervals for albuminuria at baseline and microvascular diabetic eye disease at baseline among the fifths of heart rate were estimated by logistic regression. Analyses were performed in SAS v9.2 (SAS Institute, Cary, NC), SPSS v18 (SPSS Inc., Chicago, IL), and Stata 11 (Statacorp LP, College Station, TX), and all reported *P* values are 2 sided, with the 5% threshold used to determine significance.

## Results

Of the 11 140 patients randomized into the ADVANCE study, 2 individuals did not have their baseline resting heart rate recorded. The remaining 11 138 patients are included in the present analyses. Participants had a mean age of 66 years, and 58% were male. The mean heart rate in the study population, ± standard deviation, was 74±12 beats per minute, with a range of 33 to 140 beats per minute. The characteristics of the study population according to baseline resting heart rate are shown in Table 1.

**Table 1.** Baseline Characteristics by Baseline Fifths of Heart Rate

	Total (n=11 138)	Lowest Fifth (n=2066)	2nd Fifth (n=2393)	3rd Fifth (n=2178)	4th Fifth (n=2195)	Highest Fifth (n=2306)	*P* for Trend
Heart rate, bpm, mean (range)	74 (33–140)	57 (33–63)	67 (64–70)	74 (71–76)	80 (77–83)	91 (84–140)	…

Age, y, mean±SD	65.8±6.4	67.1±6.3	65.9±6.4	65.5±6.5	65.5±6.4	65.1±6.2	<0.001

Female, n (%)	4733 (42)	664 (32)	989 (41)	973 (45)	1050 (48)	1057 (46)	<0.001

Active treatment arm for blood pressure, n (%)	5568 (50)	1024 (50)	1195 (50)	1072 (49)	1127 (51)	1150 (50)	0.56

Active treatment arm for glycemic control, n (%)	5570 (50)	1007 (49)	1216 (51)	1050 (48)	1098 (50)	1199 (52)	0.09

Systolic blood pressure, mm Hg, mean±SD	145±22	147±22	144±21	143±21	145±21	146±21	0.18

Diastolic blood pressure, mm Hg, mean±SD	81±11	79±11	80±11	81±11	81±11	82±11	<0.001

Body mass index, kg/m^2^, mean±SD	28.3±5.2	28.8±5.0	28.5±5.0	28.2±5.3	28.2±5.2	28.2±5.3	<0.001

Current cigarette smoker, n (%)	1550 (14)	234 (11)	314 (13)	333 (15)	324 (15)	345 (15)	<0.001

Regular exercise, n (%)	5112 (46)	1029 (50)	1149 (48)	994 (46)	970 (44)	970 (42)	<0.001

History of myocardial infarction, n (%)	1334 (12)	392 (19)	316 (13)	251 (12)	205 (9)	170 (7)	<0.001

History of stroke, n (%)	1022 (9)	168 (8)	197 (8)	198 (9)	217 (10)	242 (10)	<0.001

History of hospitalization for heart failure, n (%)	356 (3)	66 (3)	71 (3)	66 (3)	71 (3)	82 (4)	0.38

Previous or current atrial fibrillation, n (%)	846 (8)	199 (10)	154 (6)	128 (6)	173 (8)	192 (8)	0.68

Prior macrovascular disease, n (%)	3589 (32)	843 (41)	806 (34)	677 (31)	630 (29)	633 (27)	<0.001

Prior microvascular disease, n (%)	1152 (10)	179 (9)	249 (10)	206 (9)	242 (11)	276 (12)	<0.001

Serum total cholesterol, mmol/L, mean±SD	5.20±1.19	4.94±1.08	5.14±1.17	5.21±1.20	5.30±1.18	5.37±1.27	<0.001

Serum triglycerides, mmol/L, mean±SD	1.96±1.29	1.84±1.14	1.88±1.15	1.91±1.21	2.04±1.35	2.11±1.52	<0.001

Duration of diabetes, y, mean±SD	7.9±6.4	7.7±6.6	7.6±6.3	8.0±6.5	8.2±6.2	8.2±6.2	<0.001

Hemoglobin A1c, %, mean±SD	7.5±1.6	7.2±1.3	7.3±1.4	7.5±1.6	7.7±1.6	7.8±1.8	<0.001

Serum creatinine, μmol/L, mean±SD	87±25	91±23	87±24	85±24	84±27	86±28	<0.001

Estimated GFR, mL/min per 1.73m^2^, mean±SD	78±25	75±20	78±22	79±27	80±28	79±26	<0.001

Urinary ACR, mg/mmol, mean (IQR)	1.7 (0.8–4.5)	1.4 (0.7–3.6)	1.6 (0.7–4.1)	1.6 (0.8–3.9)	1.9 (0.9–5.2)	2.0 (0.9–5.5)	<0.001

β-Blocker, n (%)	2729 (25)	938 (45)	682 (28)	439 (20)	365 (17)	305 (13)	<0.001

Calcium channel antagonist, n (%)	3426 (31)	666 (32)	706 (30)	638 (29)	647 (29)	769 (33)	0.38

Categorical data are presented as numbers and percentages and compared with logistic regression models. Normally distributed continuous data are presented as mean ± standard deviation (SD) and are compared with a general linear model. Skewed data are presented as median and interquartile range (IQR) and are compared with a linear model after log-transformation. ACR indicates albumin:creatinine ratio; GFR, glomerular filtration rate (estimated by the Modification of Diet in Renal Disease formula). Regular exercise was defined as moderate or vigorous exercise for >15 minutes at least once per week.

### Heart Rate and Microvascular Disease at Baseline

**Figure 1.**
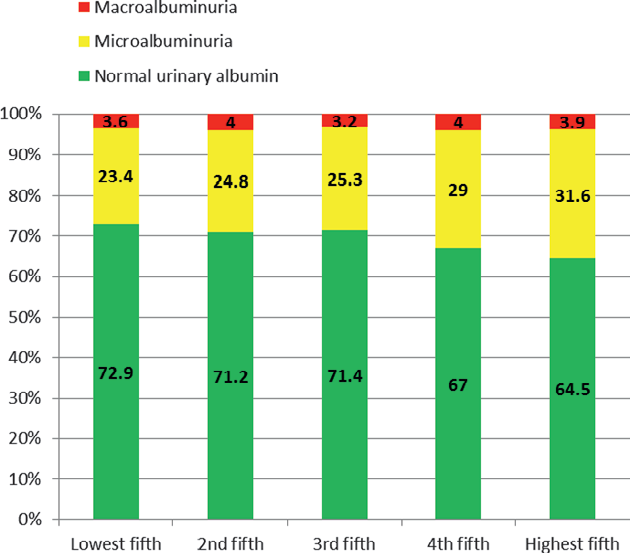
Prevalence of albuminuria according to fifths of heart rate at baseline.

At baseline, 793 patients (7.1%) had microvascular diabetic eye disease (proliferative retinopathy, retinal photocoagulation therapy, macular edema, or blindness in at least one eye thought to be due to diabetes). These individuals had a mean resting heart rate of 76±12 beats per minute, compared to 74±12 beats per minute in patients without microvascular eye disease at baseline (*P*<0.001). At the time of randomization, 7376 participants (66.2%) had normal urinary albumin levels (urinary ACR <3.4 mg/mmol [<30 μg/mg]), 2861 (25.7%) had microalbuminura (urinary ACR 3.4–33.9 mg/mmol [30–300 μg/mg]), and 401 (3.6%) had macroalbuminuria (urinary ACR >33.9 mg/mmol [>300 μg/mg]). Five hundred patients (4.5%) had missing ACR data. Figure 1 shows the prevalence of albuminuria according to baseline resting heart rate (*P* for trend <0.001). The age- and sex-adjusted and multivariable-adjusted odds ratios for (a) albuminuria and (b) microvascular diabetic eye disease at baseline according to fifth of resting heart rate (with the lowest fifth as the reference) are shown in Tables 2 and 3. These data confirm a cross-sectional association between baseline heart rate and both complications.

**Table 2.** Albuminuria at Baseline According to Fifth of Resting Heart Rate

	Adjusted for Age and Sex			Fully Adjusted*		
		
	Odds Ratio	95% Confidence Interval	*P*	Odds Ratio	95% Confidence Interval	*P*
Lowest fifth	1	Reference	…	1	Reference	…

2nd fifth	1.10	0.96–1.26	0.17	1.13	0.98–1.30	0.10

3rd fifth	1.09	0.95–1.25	0.24	1.08	0.93–1.25	0.33

4th fifth	1.34	1.17–1.54	<0.001	1.27	1.10–1.47	0.001

Highest fifth	1.50	1.31–1.71	<0.001	1.35	1.17–1.56	<0.001

* Adjusted for age, sex, body mass index, duration of diabetes, glycosylated hemoglobin, estimated glomerular filtration rate, systolic blood pressure, diastolic blood pressure, history of hospitalization for heart failure, prior macrovascular disease (see text), current cigarette smoking, participation in moderate or vigorous exercise for >15 minutes at least once weekly, total cholesterol, triglyceride level, atrial fibrillation, use of calcium channel blockers, and use of β-blockers.

**Table 3.** Microvascular Diabetic Eye Disease at Baseline According to Fifth of Resting Heart Rate

	Adjusted for Age and Sex			Fully Adjusted*		
		
	Odds Ratio	95% Confidence Interval	*P*	Odds Ratio	95% Confidence Interval	*P*
Lowest fifth	1	Reference	…	1	Reference	…

2nd fifth	1.27	0.99–1.62	0.06	1.29	0.99–1.67	0.06

3rd fifth	1.22	0.95–1.57	0.13	1.19	0.91–1.57	0.21

4th fifth	1.38	1.08–1.77	0.01	1.38	1.06–1.81	0.02

Highest fifth	1.62	1.28–2.06	<0.001	1.59	1.22–2.07	<0.001

* Adjusted for variables listed in footnote to Table 2, plus urinary albumin:creatinine ratio.

### Heart Rate and Microvascular Complications

During a median follow-up of 4.4 years, 916 patients (8.2%) experienced a major microvascular event. Three hundred and ninety-seven patients (3.6%) developed new or worsening nephropathy, and 575 (5.2%) developed new or worsening retinopathy. Fifteen patients were lost to follow-up (vital status unknown) at the end of follow-up and were censored at the last follow-up assessment. In addition, 879 patients (7.9%) died during follow-up. Patients who died before the occurrence of each outcome were censored at the time of death (786 patients for major microvascular events, 840 for retinopathy, and 817 for nephropathy were censored at the date of death).

A higher baseline resting heart rate was associated with a greater risk of developing a major microvascular complication during follow-up (Table 4). After adjustment for age, sex, and randomized treatment, a 10-beats-per-minute increase in baseline resting heart rate was associated with an 18% increase in the observed hazard. This remained after adjustment for potential confounders. The excess hazard was apparent for both components of the primary outcome (nephropathy and retinopathy) but was greater for the development of new or worsening diabetic eye disease (Table 4), where a clear linear relation is apparent. In contrast, the relation between heart rate and nephropathy is nonlinear, with the excess hazard, relative to the lowest fifth, only seen clearly in patients with a heart rate in the upper fifth (Table 4). The risk estimates were essentially unchanged even after correction for the competing risk of death (data not shown).

**Table 4.** Effects of Heart Rate on Major Microvascular Outcomes

		Adjusted for Age, Sex, and Randomized Treatment		Fully Adjusted*	
			
	No. Events/No. at Risk	HR (95% CI)	*P*	HR (95% CI)	*P*
Major microvascular events					

Heart rate per 10 bpm	916/11 138	1.18 (1.12–1.24)	<0.001	1.13 (1.07–1.20)	<0.001

Heart rate fifths					

Lowest fifth (33–63 bpm)	132/2066	1.00 (reference)		1.00 (reference)	

2nd fifth (64–70 bpm)	184/2393	1.23 (0.99–1.54)	0.07	1.27 (1.00–1.61)	0.047

3rd fifth (71–76 bpm)	161/2178	1.19 (0.94–1.49)	0.15	1.15 (0.90–1.47)	0.27

4th fifth (77–83 bpm)	194/2195	1.45 (1.16–1.81)	0.001	1.39 (1.09–1.76)	0.008

Highest fifth (84–140 bpm)	245/2306	1.78 (1.44–2.20)	<0.001	1.63 (1.29–2.06)	<0.001

*P* for trend			<0.001		<0.001

*P* for nonlinearity			0.37		0.27

New or worsening retinopathy					

Heart rate per 10 bpm	575/11 138	1.22 (1.14–1.30)	<0.001	1.16 (1.08–1.25)	<0.001

Heart rate fifths					

Lowest fifth (33–63 bpm)	71/2066	1.00 (reference)		1.00 (reference)	

2nd fifth (64–70 bpm)	96/2393	1.15 (0.84–1.56)	0.38	1.13 (0.82–1.57)	0.46

3rd fifth (71–76 bpm)	115/2178	1.51 (1.12–2.03)	0.007	1.44 (1.05–1.98)	0.02

4th fifth (77–83 bpm)	139/2195	1.82 (1.36–2.43)	<0.001	1.67 (1.23–2.28)	0.001

Highest fifth (84–140 bpm)	154/2306	1.94 (1.46–2.57)	<0.001	1.72 (1.26–2.34)	<0.001

*P* for trend			<0.001		<0.001

*P* for nonlinearity			0.70		0.69

New or worsening nephropathy					

Heart rate per 10 bpm	397/11 138	1.13 (1.04–1.22)	0.003	1.11 (1.02–1.21)	0.01

Heart rate fifths					

Lowest fifth (33–63 bpm)	69/2066	1.00 (reference)		1.00 (reference)	

2nd fifth (64–70 bpm)	93/2393	1.24 (0.91–1.70)	0.18	1.45 (1.04–2.02)	0.03

3rd fifth (71–76 bpm)	58/2178	0.86 (0.60–1.21)	0.38	0.90 (0.61–1.31)	0.57

4th fifth (77–83 bpm)	69/2195	1.05 (0.75–1.47)	0.78	1.10 (0.76–1.58)	0.61

Highest fifth (84–140 bpm)	108/2306	1.60 (1.18–2.17)	0.003	1.61 (1.15–2.26)	0.006

*P* for trend			0.02		0.05

*P* for nonlinearity			0.005		0.003

HR indicates hazard ratio; CI, confidence interval.

* Adjusted for age, sex, randomized treatment for blood pressure, randomized treatment for glucose, body mass index, duration of diabetes, glycosylated hemoglobin, prior macrovascular complication of diabetes (see text), estimated glomerular filtration rate, urinary albumin:creatinine ratio, systolic blood pressure, diastolic blood pressure, history of hospitalization for heart failure, regular exercise (see text), current cigarette smoking, total cholesterol, triglycerides, atrial fibrillation, use of calcium channel antagonists, and use of β-blockers.

**Figure 2.**
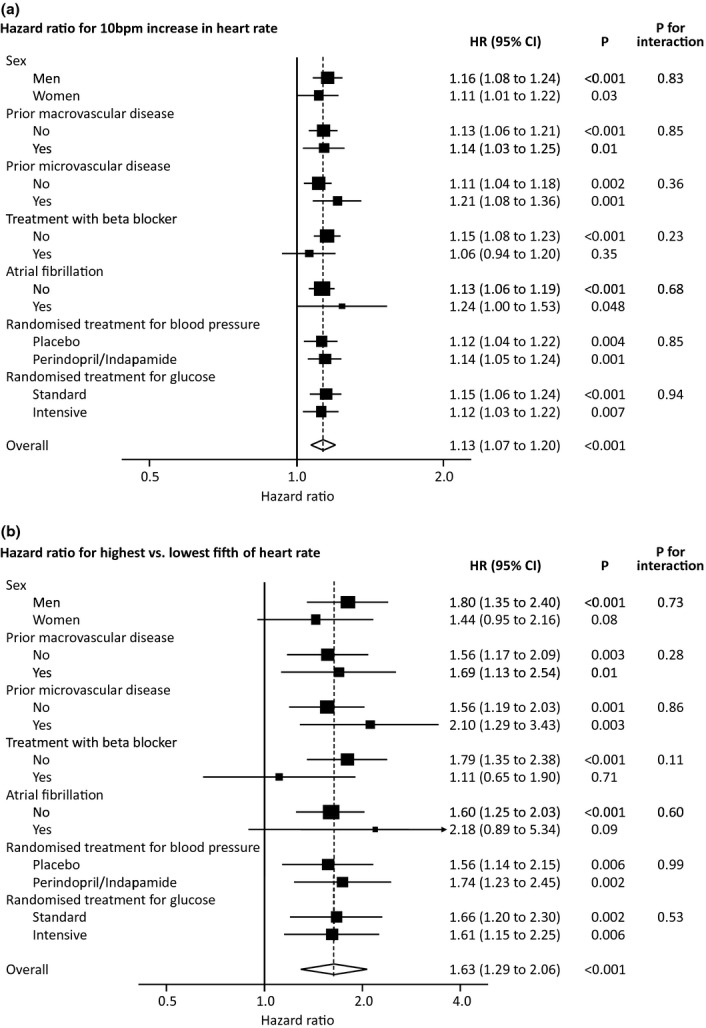
Effects of baseline heart rate on major microvascular events in subgroups. Adjusted for age, sex, randomized treatment for blood pressure, randomized treatment for glucose, body mass index, duration of diabetes, glycosylated hemoglobin, prior macrovascular complication of diabetes (see text), estimated glomerular filtration rate, urinary albumin:creatinine ratio, systolic blood pressure, diastolic blood pressure, history of hospitalization for heart failure, regular exercise (see text), current cigarette smoking, total cholesterol, triglycerides, atrial fibrillation, use of calcium channel antagonists, and use of β-blockers. Where relevant, the variable used for subgrouping was excluded from the model. Centers of boxes are placed at the estimates of adjusted hazard ratio (HR) for each subgroup. Areas of the boxes are proportional to the reciprocal of the variance of the estimates. Horizontal lines represent 95% confidence intervals (CI). Vertical broken lines indicate the estimate of overall adjusted hazard ratio. The widths of diamonds represent 95% CI. bpm indicates beats per minute.

The increased risk of microvascular complications associated with a higher heart rate was observed in both sexes, in patients who had suffered a prior major macrovascular complication (myocardial infarction, stroke, hospital admission for a transient ischemic attack, hospital admission for angina, coronary revascularization, peripheral revascularization, or amputation secondary to peripheral vascular disease) and in those who had not (Figure 2). Similarly, an excess hazard was seen in patients both with and without preexisting microvascular disease (Figure 2). It also was observed in patients who were not on a β-blocker but not in those who were on a β-blocker (Figure 2), although there was no statistical evidence of an interaction. A higher heart rate was associated with a greater risk of a microvascular complication both in patients who were in sinus rhythm and those with atrial fibrillation (on an electrocardiogram performed either at baseline or previously^22^).

## Discussion

The present study demonstrates that a higher resting heart rate predicts a greater risk of future microvascular complications in patients with type 2 diabetes mellitus. As far as we are aware, this has not been shown previously. The excess hazard associated with a higher heart rate is independent of many other factors that can influence heart rate and affect outcome in this setting. The association is particularly apparent for the occurrence or worsening of diabetic eye disease but is also evident for the occurrence or worsening of diabetic nephropathy.

### Heart Rate and Microvascular Events

The relation between higher heart rates and major adverse cardiac events, such as myocardial infarction, cardiac death, and hospitalization with heart failure, is now well recognized.^23^ Indeed, we recently reported in ADVANCE that a higher heart rate was a strong and independent predictor of all-cause death and major cardiovascular complications, including cardiovascular death, myocardial infarction, and stroke.^11^ In contrast, there are limited prior data, almost entirely cross sectional, assessing the relation between heart rate and microvascular disease. In patients with hypertension, Bohm and colleagues^12^ reported that a higher heart rate is associated with increased prevalence and severity of microalbuminuria. This relation was observed regardless of treatment with β-blockers^12^ and irrespective of whether the patient had a history of atrial fibrillation.^13^ However, in a recently reported analysis of The PROspective pioglitAzone Clinical Trial In macroVascular Events, a multicenter double-blind assessment of the effects of pioglitazone versus placebo on major cardiovascular events in patients with type 2 diabetes and a history of macrovascular disease, no association was observed between resting heart rate and microalbuminuria.^15^ Despite this, heart rate and microalbuminuria were each independently predictive of the composite endpoint of all-cause death, myocardial infarction, and stroke in this study.^15^

Neither of the aforementioned studies^13,15^ assessed the relation between heart rate and incident albuminuria. Two recent prospective studies have, however, identified a higher heart rate as a predictor of renal dysfunction in the general population.^24,25^ In the Atherosclerosis Risk in Communities (ARIC) Study, the hazard of developing end-stage renal disease was almost doubled among patients with a resting heart rate in the upper quarter, when compared to the remaining subjects, during a median of 16 years' follow-up.^24^ Similarly, in a cohort of subjects participating in a Japanese health screening program, a higher heart rate was associated with a greater risk of declining estimated glomerular filtration rate and of developing dipstick proteinuria (≥1) during 5 years of follow-up.^25^

An association between a higher resting heart rate and prevalent retinopathy in patients with diabetes has been reported previously,^16^ although not consistently.^26,27^ In the Wisconsin Epidemiological Study of Diabetic Retinopathy, although patients with a higher resting heart rate had a greater risk of incident macular edema and progressive diabetic retinopathy over a 4-year period, this was attenuated and was nonsignificant after correction for other factors, including age, duration of diabetes, glycosylated hemoglobin, and blood pressure.^28^ In contrast, in our much larger cohort, followed up for 4.4 years, we have observed strong and independent associations between resting heart rate and incident and progressive diabetic eye disease, even after correction for a wide range of potential confounding factors (including all of those mentioned above). Apart from differences in power, the discrepant results could reflect variances in the study populations and protocols. The Wisconsin Epidemiological Study of Diabetic Retinopathy recruited a community-based cohort with a high prevalence of younger, insulin-requiring patients, and the incidence and progression of retinopathy was determined with fundus photography.

### Potential Mechanisms

Although a higher resting heart rate predicts a greater prevalence of albuminuria and diabetic eye disease at baseline and higher risk of subsequent microvascular events, the results could reflect an association between heart rate and other factors that might mediate microvascular disease. There are, however, several plausible mechanisms by which an elevated heart rate might predispose to such complications.

It has been suggested that a higher heart rate might promote microalbuminuria because of increased exposure of the glomerulus to arterial pressure waves.^12^ A similar mechanism also might explain the relation between more rapid heart rates and ocular disease. An increased heart rate also has a variety of direct detrimental cardiovascular consequences. These include unfavorable effects on endothelial function and proatherosclerotic activity,^29^ which are important factors in the progression of nephropathy and retinopathy.^30–32^ A higher heart rate also is associated with factors such as obesity, higher blood pressure, a proatherosclerotic lipid profile, and reduced physical activity,^10,33^ all of which are associated with an increased risk of microvascular complications and are targets for intervention to improve outcome in patients with diabetes mellitus.^34^ Finally, a faster resting heart rate is a characteristic feature of autonomic neuropathy, which is in turn associated with an increased prevalence of other complications, such as nephropathy and retinopathy.^35^ It is possible, therefore, that the observed relation between heart rate and microvascular events is mediated at least in part by sympathetic overactivity, although correlations between heart rate and measures of sympathetic tone are relatively weak.^36^

When baroreflex control of heart rate is assessed by sensitive methods, abnormalities are apparent at an early stage in patients with diabetes. These changes predate obvious diabetic complications and evidence of autonomic dysfunction that can be detected by conventional methods.^37,38^ Intriguingly, a higher heart rate per se also has been shown to predict an increased risk of developing incident diabetes.^39^ This could reflect physical deconditioning and other factors that predispose to developing diabetes but reinforces the complex interrelations among heart rate, neural mechanisms, and metabolic control.

It could be that the observed hazard associated with a higher heart rate is due to a combination of mechanisms and that the importance of each varies among individuals. Likewise, the mechanisms could have synergistic effects. For example, a higher heart rate might result in direct microvascular damage, but this could be accentuated by the presence of hypertension. In particular, elevated nocturnal blood pressure in diabetes is associated with incident microalbuminuria^40^ and progressive nephropathy.^41^ The risk attributable to this “nondipping” pattern might not be conveyed in the measured daytime blood pressure and might be magnified by a higher resting heart rate, which increases exposure of the microvasculature to the elevated pressures.

In summary, the mechanisms by which a higher resting heart rate predicts an increased risk of microvascular complications in type 2 diabetes mellitus are unclear, and it could be that several interrelated processes are involved. One potential method of assessing whether there might be a direct relation would be to assess the effects of a pure rate-limiting drug, such as ivabradine, on microvascular outcomes.

### Differences in the Observed Relation Between Heart Rate and Eye Disease and Nephropathy

In this analysis, the relation between resting heart rate and the risk of developing diabetic eye disease seems stronger than the association with incident nephropathy. In addition, in contrast to retinopathy, the data suggest that the relation between heart rate and renal disease is nonlinear, with the excess hazard clearly seen only in patients with a resting heart rate in the upper fifth. Similarly, in the baseline, cross-sectional data, the increased prevalence of microalbuminuria is most obvious in patients with heart rates in the upper two fifths. This raises the possibility that the relation between heart rate and microvascular disease could vary in differing vascular beds. This is not unexpected, given that the effects of blood pressure–lowering treatment and more intensive glycemic control in the ADVANCE study were more apparent in the kidney than in the eye. Similar differential effects of treatment on differing microvascular complications have been observed in other large studies.^42,43^ Whether this reflects differing pathophysiological mechanisms, is a chance finding, or is due to the definitions used and the relatively short duration of follow-up is not clear.

### Strengths and Limitations

The present study explores the relation between heart rate and future microvascular complications in patients with type 2 diabetes mellitus in a large population recruited from 20 countries and with diverse ethnic backgrounds, which was well characterized and followed up closely. The primary endpoint of this epidemiological evaluation was a coprimary endpoint of the main ADVANCE study, and all components of it were independently adjudicated according to predefined criteria. Nevertheless, it has the limitations inherent in any such post hoc analysis. Although the large study population ensures considerable statistical power and allows reliable correction for many potential confounding factors, it is not possible to fully correct for all possible confounders. In particular, autonomic function was not assessed at baseline.

## Conclusion

Patients with type 2 diabetes mellitus who have a higher resting heart rate have a greater prevalence of microalbuminuria and diabetic eye disease. They also experience a greater incidence of new-onset or progressive nephropathy and retinopathy, which suggests that an increased heart rate might be a potential mediator of these outcomes. Further work is required to assess this and to determine whether lifestyle measures or pharmacological therapies that can slow heart rate might reduce the risk of microvascular complications. In particular, the benefits of heart rate reduction in the prevention of progressive diabetic retinopathy deserve further attention.

## References

[1] The effect of intensive treatment of diabetes on the development and progression of long-term complications in insulin-dependent diabetes mellitus. The Diabetes Control and Complications Trial research group. N Engl J Med. 1993;329:977-986. 8366922 10.1056/NEJM199309303291401

[2] Rajala U Pajunpaa H Koskela P Keinanen-Kiukaanniemi S High cardiovascular disease mortality in subjects with visual impairment caused by diabetic retinopathy. Diabetes Care. 2000;23:957-961. 10895846 10.2337/diacare.23.7.957

[3] Klein R Klein BE Moss SE Cruickshanks KJ Association of ocular disease and mortality in a diabetic population. Arch Ophthalmol. 1999;117:1487-1495. 10565517 10.1001/archopht.117.11.1487

[4] Gerstein HC Mann JF Yi Q Zinman B Dinneen SF Hoogwerf B Halle JP Young J Rashkow A Joyce C Nawaz S Yusuf S Albuminuria and risk of cardiovascular events, death, and heart failure in diabetic and nondiabetic individuals. JAMA. 2001;286:421-426. 11466120 10.1001/jama.286.4.421

[5] McClintic BR McClintic JI Bisognano JD Block RC The relationship between retinal microvascular abnormalities and coronary heart disease: a review. Am J Med. 2010;123:374.e1-374.e7. 10.1016/j.amjmed.2009.05.030 PMC2922900 20362758

[6] Arnlov J Evans JC Meigs JB Wang TJ Fox CS Levy D Benjamin EJ D'Agostino RB Vasan RS Low-grade albuminuria and incidence of cardiovascular disease events in nonhypertensive and nondiabetic individuals: the Framingham Heart Study. Circulation. 2005;112:969-975. 16087792 10.1161/CIRCULATIONAHA.105.538132

[7] Klausen K Borch-Johnsen K Feldt-Rasmussen B Jensen G Clausen P Scharling H Appleyard M Jensen JS Very low levels of microalbuminuria are associated with increased risk of coronary heart disease and death independently of renal function, hypertension, and diabetes. Circulation. 2004;110:32-35. 15210602 10.1161/01.CIR.0000133312.96477.48

[8] Romundstad S Holmen J Hallan H Kvenild K Ellekjaer H Microalbuminuria and all-cause mortality in treated hypertensive individuals: does sex matter? The Nord-Trondelag Health Study (HUNT), Norway. Circulation. 2003;108:2783-2789. 14623803 10.1161/01.CIR.0000103667.27493.32

[9] Fox K Borer JS Camm AJ Danchin N Ferrari R Lopez Sendon JL Steg PG Tardif JC Tavazzi L Tendera M Resting heart rate in cardiovascular disease. J Am Coll Cardiol. 2007;50:823-830. 17719466 10.1016/j.jacc.2007.04.079

[10] Palatini P Elevated heart rate in cardiovascular diseases: a target for treatment?. Prog Cardiovasc Dis. 2009;52:46-60. 19615493 10.1016/j.pcad.2009.05.005

[11] Hillis GS Woodward M Rodgers A Chow CK Li Q Zoungas S Patel A Webster R Batty GD Ninomiya T Mancia G Poulter NR Chalmers J Resting heart rate and the risk of death and cardiovascular complications in patients with type 2 diabetes mellitus. Diabetologia. 2012;55:1283-1290. 22286552 10.1007/s00125-012-2471-y PMC4170780

[12] Bohm M Reil JC Danchin N Thoenes M Bramlage P Volpe M Association of heart rate with microalbuminuria in cardiovascular risk patients: data from i-SEARCH. J Hypertens. 2008;26:18-25. 18090536 10.1097/HJH.0b013e3282f05c8a

[13] Bohm M Thoenes M Neuberger HR Graber S Reil JC Bramlage P Volpe M Atrial fibrillation and heart rate independently correlate to microalbuminuria in hypertensive patients. Eur Heart J. 2009;30:1364-1371. 19383737 10.1093/eurheartj/ehp124

[14] Ritz E Viberti GC Ruilope LM Rabelink AJ Izzo JL Jr Katayama S Ito S Mimran A Menne J Rump LC Januszewicz A Haller H Determinants of urinary albumin excretion within the normal range in patients with type 2 diabetes: the Randomised Olmesartan And Diabetes MicroAlbuminuria Prevention (ROADMAP) study. Diabetologia. 2010;53:49-57. 19876613 10.1007/s00125-009-1577-3 PMC2789932

[15] Pfister R Erdmann E Schneider CA Association and prognostic impact of heart rate and micro-albuminuria in patients with type 2 diabetes and cardiovascular disease: results from the PROactive trial. J Atheroscler Thromb. 2011;18:65-71. 21088369 10.5551/jat.6247

[16] Imano E Miyatsuka T Motomura M Kanda T Matsuhisa M Kajimoto Y Yamasaki Y Hori M Heart rate elevation and diabetic retinopathy in patients with type 2 diabetes mellitus and normoalbuminuria. Diabetes Res Clin Pract. 2001;52:185-191. 11323088 10.1016/s0168-8227(01)00219-4

[17] Patel A MacMahon S Chalmers J Neal B Woodward M Billot L Harrap S Poulter N Marre M Cooper M Glasziou P Grobbee DE Hamet P Heller S Liu LS Mancia G Mogensen CE Pan CY Rodgers A Williams B Effects of a fixed combination of perindopril and indapamide on macrovascular and microvascular outcomes in patients with type 2 diabetes mellitus (the ADVANCE trial): a randomised controlled trial. Lancet. 2007;370:829-840. 17765963 10.1016/S0140-6736(07)61303-8

[18] Patel A MacMahon S Chalmers J Neal B Billot L Woodward M Marre M Cooper M Glasziou P Grobbee D Hamet P Harrap S Heller S Liu L Mancia G Mogensen CE Pan C Poulter N Rodgers A Williams B Bompoint S de Galan BE Joshi R Travert F Intensive blood glucose control and vascular outcomes in patients with type 2 diabetes. N Engl J Med. 2008;358:2560-2572. 18539916 10.1056/NEJMoa0802987

[19] Study rationale and design of advance: Action in Diabetes and Vascular Disease—Preterax and Diamicron MR Controlled Evaluation. Diabetologia. 2001;44:1118-1120. 11596665 10.1007/s001250100612

[20] Woodward M Epidemiology: Study Design and Data Analysis. 2005 2nd ed. Boca Raton, FL Chapman and Hall/CRC Press 554-557.

[21] Fine JP Gray RJ A proportional hazards model for the subdistribution of a competing risk. J Am Stat Assoc. 1999;94:496-509.

[22] Du X Ninomiya T de Galan B Abadir E Chalmers J Pillai A Woodward M Cooper M Harrap S Hamet P Poulter N Lip GY Patel A Risks of cardiovascular events and effects of routine blood pressure lowering among patients with type 2 diabetes and atrial fibrillation: results of the ADVANCE study. Eur Heart J. 2009;30:1128-1135. 19282274 10.1093/eurheartj/ehp055

[23] Fox KM Current status: heart rate as a treatable risk factor. Eur Heart J. 2011;13 suppl C C30-C36.

[24] Brotman DJ Bash LD Qayyum R Crews D Whitsel EA Astor BC Coresh J Heart rate variability predicts ESRD and CKD-related hospitalization. J Am Soc Nephrol. 2010;21:1560-1570. 20616169 10.1681/ASN.2009111112 PMC3013524

[25] Inoue T Iseki K Iseki C Ohya Y Kinjo K Takishita S Heart rate as a risk factor for developing chronic kidney disease: longitudinal analysis of a screened cohort. Clin Exp Nephrol. 2009;13:487-493. 19444548 10.1007/s10157-009-0193-3

[26] Klein R Klein BE Moss SE Davis MD DeMets DL The Wisconsin Epidemiologic Study of Diabetic Retinopathy, II. Prevalence and risk of diabetic retinopathy when age at diagnosis is less than 30 years. Arch Ophthalmol. 1984;102:520-526. 6367724 10.1001/archopht.1984.01040030398010

[27] Klein R Klein BE Moss SE Davis MD DeMets DL The Wisconsin Epidemiologic Study of Diabetic Retinopathy, III. Prevalence and risk of diabetic retinopathy when age at diagnosis is 30 or more years. Arch Ophthalmol. 1984;102:527-532. 6367725 10.1001/archopht.1984.01040030405011

[28] Wong TY Moss SE Klein R Klein BE Is the pulse rate useful in assessing risk of diabetic retinopathy and macular oedema? The Wisconsin Epidemiological Study of Diabetic Retinopathy. Br J Ophthalmol. 2001;85:925-927. 11466246 10.1136/bjo.85.8.925 PMC1724060

[29] Ceconi C Guardigli G Rizzo P Francolini G Ferrari R The heart rate story. Eur Heart J. 2011;13 suppl C C4-C13.

[30] Nakagawa T Tanabe K Croker BP Johnson RJ Grant MB Kosugi T Li Q Endothelial dysfunction as a potential contributor in diabetic nephropathy. Nat Rev Nephrol. 2011;7:36-44. 21045790 10.1038/nrneph.2010.152 PMC3653134

[31] Stehouwer CD Endothelial dysfunction in diabetic nephropathy: state of the art and potential significance for non-diabetic renal disease. Nephrol Dial Transplant. 2004;19:778-781. 15031329 10.1093/ndt/gfh015

[32] Rosenson RS Fioretto P Dodson PM Does microvascular disease predict macrovascular events in type 2 diabetes?. Atherosclerosis. 2011;218:13-18. 21763654 10.1016/j.atherosclerosis.2011.06.029

[33] Jurca R Jackson AS LaMonte MJ Morrow JR Jr Blair SN Wareham NJ Haskell WL van Mechelen W Church TS Jakicic JM Laukkanen R Assessing cardiorespiratory fitness without performing exercise testing. Am J Prev Med. 2005;29:185-193. 10.1016/j.amepre.2005.06.004 16168867

[34] Standards of medical care in diabetes—2008. Diabetes Care. 2008;31 suppl 1 S12-S54. 18165335 10.2337/dc08-S012

[35] Vinik AI Maser RE Mitchell BD Freeman R Diabetic autonomic neuropathy. Diabetes Care. 2003;26:1553-1579. 12716821 10.2337/diacare.26.5.1553

[36] Grassi G Vailati S Bertinieri G Seravalle G Stella ML Dell'Oro R Mancia G Heart rate as marker of sympathetic activity. J Hypertens. 1998;16:1635-1639. 9856364 10.1097/00004872-199816110-00010

[37] Frattola A Parati G Gamba P Paleari F Mauri G Di Rienzo M Castiglioni P Mancia G Time and frequency domain estimates of spontaneous baroreflex sensitivity provide early detection of autonomic dysfunction in diabetes mellitus. Diabetologia. 1997;40:1470-1475. 9447956 10.1007/s001250050851

[38] Weston PJ James MA Panerai R McNally PG Potter JF Thurston H Swales JD Abnormal baroreceptor-cardiac reflex sensitivity is not detected by conventional tests of autonomic function in patients with insulin-dependent diabetes mellitus. Clin Sci (Lond). 1996;91:59-64. 8774261 10.1042/cs0910059

[39] Carnethon MR Golden SH Folsom AR Haskell W Liao D Prospective investigation of autonomic nervous system function and the development of type 2 diabetes: the Atherosclerosis Risk in Communities study, 1987–1998. Circulation. 2003;107:2190-2195. 12695289 10.1161/01.CIR.0000066324.74807.95

[40] Lurbe E Redon J Kesani A Pascual JM Tacons J Alvarez V Batlle D Increase in nocturnal blood pressure and progression to microalbuminuria in type 1 diabetes. N Engl J Med. 2002;347:797-805. 12226150 10.1056/NEJMoa013410

[41] Knudsen ST Laugesen E Hansen KW Bek T Mogensen CE Poulsen PL Ambulatory pulse pressure, decreased nocturnal blood pressure reduction and progression of nephropathy in type 2 diabetic patients. Diabetologia. 2009;52:698-704. 19183937 10.1007/s00125-009-1262-6

[42] Duckworth W Abraira C Moritz T Reda D Emanuele N Reaven PD Zieve FJ Marks J Davis SN Hayward R Warren SR Goldman S McCarren M Vitek ME Henderson WG Huang GD Glucose control and vascular complications in veterans with type 2 diabetes. N Engl J Med. 2009;360:129-139. 19092145 10.1056/NEJMoa0808431

[43] Ismail-Beigi F Craven T Banerji MA Basile J Calles J Cohen RM Cuddihy R Cushman WC Genuth S Grimm RH Jr Hamilton BP Hoogwerf B Karl D Katz L Krikorian A O'Connor P Pop-Busui R Schubart U Simmons D Taylor H Thomas A Weiss D Hramiak I Effect of intensive treatment of hyperglycaemia on microvascular outcomes in type 2 diabetes: an analysis of the ACCORD randomised trial. Lancet. 2010;376:419-430. 20594588 10.1016/S0140-6736(10)60576-4 PMC4123233

